# Dietary Intake, Nutritional Adequacy, and Food Sources of Protein and Relationships with Personal and Family Factors in Spanish Children Aged One to <10 Years: Findings of the EsNuPI Study †

**DOI:** 10.3390/nu13041062

**Published:** 2021-03-24

**Authors:** Casandra Madrigal, María José Soto-Méndez, Ángela Hernández-Ruiz, Teresa Valero, Federico Lara Villoslada, Rosaura Leis, Emilio Martínez de Victoria, José Manuel Moreno, Rosa M. Ortega, María Dolores Ruiz-López, Gregorio Varela-Moreiras, Ángel Gil

**Affiliations:** 1 Department of Nutrition and Food Science, Faculty of Pharmacy, University of Granada, 18071 Granada, Spain; casandram@correo.ugr.es; 2 Iberoamerican Nutrition Foundation (FINUT), Armilla, 18016 Granada, Spain; msoto@finut.org (M.J.S.-M.); ahernandez@finut.org (Á.H.-R.); agil@ugr.es (Á.G.); 3 Spanish Nutrition Foundation (FEN), 28010 Madrid, Spain; tvalero@fen.org.es (T.V.); gvarela@ceu.es (G.V.-M.); 4 Instituto de Nutrición Puleva, 18004 Granada, Spain; federico.lara@lactalis.es; 5 Unit of Pediatric Gastroenterology, Department of Pediatrics, Hepatology and Nutrition University Clinical Hospital of Santiago, 15706 Santiago de Compostela, Spain; mariarosaura.leis@usc.es; 6 Health Research Institute of Santiago (IDIS), University of Santiago de Compostela, 15706 Santiago de Compostela, Spain; 7 Physiopathology of Obesity and Nutrition (CIBEROBN), Instituto de Salud Carlos III (ISCIII), 28029 Madrid, Spain; 8 Department of Physiology, Faculty of Pharmacy, University of Granada, 18071 Granada, Spain; emiliom@ugr.es; 9 Biomedical Research Center, Institute of Nutrition and Food Technology “José Mataix”, University of Granada, 18100 Granada, Spain; 10 Pediatric Department, University of Navarra Clinic, 28027 Madrid, Spain; jmorenov@unav.es; 11 Department of Nutrition and Food Science, Faculty of Pharmacy, Complutense University of Madrid, 28040 Madrid, Spain; rortega@ucm.es; 12 Department of Pharmaceutical and Health Sciences, Faculty of Pharmacy, CEU San Pablo University, 28668 Madrid, Spain; 13 Department of Biochemistry and Molecular Biology II, University of Granada, 18071 Granada, Spain

**Keywords:** dietary proteins, proteins, dietary animal protein, dietary plant protein, pediatric nutrition, fortified milk, food sources, dairy products, Spanish children, EsNuPI study

## Abstract

Diet in the first years of life is an important factor in growth and development. Dietary protein is a critical macronutrient that provides both essential and nonessential amino acids required for sustaining all body functions and procedures, providing the structural basis to maintain life and healthy development and growth in children. In this study, our aim was to describe the total protein intake, type and food sources of protein, the adequacy to the Population Reference Intake (PRI) for protein by the European Food Safety Authority (EFSA), and the Recommended Dietary Allowance (RDA) by the Institute of Medicine (IoM). Furthermore, we analyzed whether the consumption of dairy products (including regular milk, dairy products, or adapted milk formulas) is associated with nutrient adequacy and the contribution of protein to diet and whole dietary profile in the two cohorts of the EsNuPI (in English, Nutritional Study in the Spanish Pediatric Population) study; one cohort was representative of the Spanish population from one to <10 years old (*n* = 707) (Spanish reference cohort, SRS) who reported consuming all kinds of milk and one was a cohort of the same age who reported consuming adapted milk over the last year (including follow-on formula, growing up milk, toddler’s milk, and enriched and fortified milks) (*n* = 741) (adapted milk consumers cohort, AMS). The children of both cohorts had a high contribution from protein to total energy intake (16.79% SRS and 15.63% AMS) and a high total protein intake (60.89 g/day SRS and 53.43 g/day AMS). We observed that protein intake in Spanish children aged one to <10 years old was above the European and international recommendations, as well as the recommended percentages for energy intakes. The main protein sources were milk and dairy products (28% SRS and 29% AMS) and meat and meat products (27% SRS and 26% AMS), followed by cereals (16% SRS and 15% AMS), fish and shellfish (8% in both cohorts), eggs (5% SRS and 6% AMS), and legumes (4% in both cohorts). In our study population, protein intake was mainly from an animal origin (meat and meat products, milk and dairy products, fish and shellfish, and eggs) rather than from a plant origin (cereals and legumes). Future studies should investigate the long-term effect of dietary protein in early childhood on growth and body composition, and whether high protein intake affects health later in life.

## 1. Introduction

Diet in early childhood plays an important role in growth and development. Multiple studies have reported different macro- and micronutrients’ roles in a linear growth trajectory [1]. In particular, dietary protein is a critical macronutrient, because it provides both the essential and nonessential amino acids necessary to provide the fundamental basis to sustain life and the healthy development of children [1,2]. The amount and quality of protein are important for ideal growth in the first years of life; for that reason, the consumption of low-quality protein could lead to stunting and/or wasting [3].

Current protein requirements for all age groups are defined by the European Food Safety Authority (EFSA) and Institute of Medicine (IoM) [2,4]. The EFSA recommendations for protein are expressed as the Population Reference Intake (PRI), which is the level of nutrient intake that is adequate for virtually all people in a population group (PRIs, expressed in grams of protein/kilogram body weight/day (g/kg/day)). These recommendations range from 0.85 to 1.14, depending on the age groups.

The protein recommendations from the IoM [2], reported as the Recommended Dietary Allowance (RDA), indicate the specific nutrient intake requirements that the majority of children in a population need for good health and growth, depending on their age and sex; the RDA ranges from 0.95 to 1.05, and is also expressed in g of protein/kg body weight/day (g/kg/day).

Protein intake lower than these recommended ranges could lead to inadequate growth [5]. Several studies have found that a lower percentage of energy derived from dietary protein intake had a strong association with lower length for age [6].

In recent decades, there has been a global transition in most developed countries towards higher protein diets that strongly exceed the recommended intakes. Recent studies claim for potentially unfavorable effects due to increased consumption of proteins in childhood. In particular, excess protein intake during early life might result in several non-communicable diseases later in adulthood [7].

The most recent information on Spanish infants and children reported higher protein intake than the available recommendations [8,9,10]. The “Alimentando la Salud del Mañana” (ALSALMA) study (*n* = 1320) was developed in children aged 0–36 months, finding that 95.9% of the children between seven and 36 months reported an intake more than twice the RDA for protein [8]. Furthermore, the Anthropometry, Intake, and Energy Balance in Spain (ANIBES) study reported that the protein intake in children 9 to 12 years old (*n* = 213) was above the upper recommended limit (15% energy intake (EI)) established by the Nutritional Objectives for the Spanish population [11]. The mean dietary protein intake was 77.6 g, representing roughly 16% of EI [9]. Finally, when using the Acceptable Macronutrient Distribution Range (AMDR) as a reference, the National Dietary Survey on the Child and Adolescent Population project in Spain (ENALIA) (*n* = 1862) concluded that, for children aged 1–3 years, protein contribution to energy was 16.7%, and for children aged 4–8 years, it was 17.1% [10].

Similar results have been presented in other countries. The National Health and Nutrition Examination Survey 2001–2014 (NHANES) (*n* = 15,829; aged two to 80) concluded that most of the United States population exceeds the minimum recommendations for protein intake, but they remain clearly below the tolerable upper limit of the AMDR (14% to 16% EI) [12]. Brunner et al. reported that the protein intake was three to four times the Recommended Daily Intake (RDI) and reached the UL of 15% of total EI in Swiss toddlers between one to three years (*n* = 188) [13]. On the contrary, in the Identification and Prevention of Dietary- and Lifestyle-Induced Health Effects in Children and Infants (IDEFICS) research project (*n* = 9560; two to nine years old), the majority of European children met the protein intake recommended in the D-AC-H reference values (58.6 g/day) [14].

Some studies conclude that the specific sources of protein, and not the total protein intake, can cause accelerated growth, and may have diverse metabolic effects with consequences for growth in the early years due to their amino acid composition [15]. The intake of animal protein, especially from dairy, has a stronger positive association with growth than plant protein, and bone density in childhood is positively associated with increased protein intake [16,17].

For these reasons, it is important to investigate the protein intake, type of protein, source of the protein, adequacy in terms of European and international recommendations, and influences of sociodemographic factors in Spanish children. Furthermore, the Nutritional Study in the Spanish Pediatric Population (EsNuPI) study was designed to determine the dietary patterns, physical activity, and sedentary behaviors of Spanish children from one to <10 years old in urban areas (>50,000 inhabitants) distributed across nine geographical areas [18].

In Spain and other developed countries, a high quantity of adapted milk formulas (follow-on and toddler’s milk) and enriched and fortified milk for children and infants are consumed [18]. In this type of milk, the protein content is usually lower than in cow’s milk; therefore, it is interesting to know how the intake of these products affects the intake of nutrients.

Briefly, in the EsNuPI study, two cohorts were selected, one Spanish reference cohort (SRS) and one adapted milk consumers cohort (AMS). The SRS cohort drank all types of milk, and the AMS cohort regularly consumed adapted milk (including follow-on formula, growing up milk, toddler’s milk, and enriched and fortified milks).

Therefore, the objectives of the present study were: (1) to describe the dietary protein intakes in the SRS and AMS cohorts divided into three age groups; (2) to evaluate the adequacy to the EFSA and IoM dietary allowances, and with the cutoff points established in our study, evaluate the reference population (SRS) for total protein intake; (3) to describe the food sources providing total, animal, and plant protein intake; (4) to investigate whether the consumption of dairy products (including the standard milk, dairy products, or adapted milks) is associated with nutrient adequacy in children, with a diverse contribution of protein to the dietary intake, and with the whole dietary profile; and (5) to evaluate the influences of a number of sociodemographic aspects on total, animal, and plant protein intake.

## 2. Materials and Methods

### 2.1. Study Design and Population

The information and data reported in this document were collected as part of the EsNuPI study, a cross-sectional research study conducted from October 2018 to January 2019. The study protocol and methodology have been previously reported [18].

The EsNuPI study collected information about the dietary and nutrient intake, dietary patterns, physical activity, and sedentary behaviors of non-vegan one to <10 years old Spanish children living in urban areas, distributed in nine regions, according to Nielsen Spanish areas.

A total of 1514 children agreed to participate in the study, and 742 children aged one to <10 years old were randomly selected, called the Spanish reference cohort, SRS, who regularly consumed all types of milk e.g., cow, goat, other mammal milk, and fortified and adapted milk formulas (including follow-on formula, toddler’s milk, growing up milk, and fortified and enriched milks) in the last 12 months, and 772 children of the same age called the adapted milk consumer cohort, AMS, who only consumed adapted milk formulas over the last year. Finally, 1448 (95.6%) children whose parents or caregivers signed a consent form and concluded the second 24-h dietary recall (24-h DR) (707 SRS and 741 AMS), and all of the questionnaires of the study were enrolled; details of the subject characteristics have been published elsewhere [18].

The SRS cohort represented 48.8% and the AMS 51.1%. The cohorts were stratified by three age groups: group 1-Gp 1, one to <3 years old (31.5%); group 2-Gp 2, three to <6 years old (34.9%); and group 3-Gp 3, six to <10 years old (33.6%).

### 2.2. Dietary Data Assessment

The EFSA European Union (EU) Menu Project guidance was followed to conduct the dietary data collection [19].

In the EsNuPI study, the dietary intake of children was assessed using two 24-h DRs (one face-to-face and one telephone food recall) of nonconsecutive days (including one weekend day), asking the parents or caregivers to be facilitators to determine the children’s dietary intake when necessary.

Children themselves and/or their caregivers described the dietary intake (ingredients, different brands, and cooking methods) in detail; the interviewer used the provided information for the appropriate coding and weight assignment for each food item. For foods prepared at home, each ingredient was introduced separately. Some standard recipes were disaggregated into their components using nutrient information from the database.

As support material, the interviewer applied the tables of common home measures, and habitual portion sizes for Spain population [20,21] and the photo guide of common portions sizes of Spanish foods, including 204 food items regularly consumed by the Spanish population [22].

The nutritionists of the study examined the dietary information obtained by the interviewers and requested review when necessary. Average daily EI (kcal) and macronutrient intakes (grams) were calculated using the software VD-FEN 2.1, a dietary evaluation program designed by the Spanish Nutrition Foundation (FEN), mainly based on data from a Spanish food composition table [20].

In order to determine the protein contribution from different foods, the 746 food items recorded by children in the two 24-h DRs were classified into 18 food groups of similar nutrient content. Further disaggregation of the food items was performed to allow classification into animal, plant, and mixed protein sources.

Animal protein was defined as protein in g/day and the percentage of total energy from proteins, sourced mainly from meat and meat products, milk and dairy products, eggs, fish and shellfish, and other dairy products. Protein intake from cereals, fruit, vegetable, nuts, legumes, and condiments was defined as plant protein. Mixed protein came mainly from the following sources: bakery and pastry, chocolate, ready to cook/eat, appetizers, and sauces. The group of oils and fats was not classified as a source of protein.

Individual usual intakes (IUIs) of total protein were used to determine the adequacy of the dietary reference values according to PRI for protein by EFSA and the RDA by IoM recommendations.

We have used the EFSA recommendations, as we have followed the European methodology to collect information for the development of the EsNuPI study; this allowed us to compare our research results with other Spanish and European studies and the IoM recommendations with other international studies.

P25 and P75 of the IUI of total protein intake according to the age groups was estimated in the SRS cohort to define the cutoff points that allow us to estimate the percentage of children with a below or above protein intake according to the distribution of our own data set.

Protein intake is presented in four approaches: (1) grams of protein per day, (2) grams of protein per kilogram of body weight, (3) the percentage of energy from protein, and (4) intakes of animal, plant, and mixed protein.

Finally, we evaluated specifically eight different food groups to determine the percentage of contribution to the total animal and plant protein intake: (1) milk and dairy products (including cow’s milk, adapted milks (follow-on, toddler’s, growing up, and fortified or enriched milk formulas), yogurt, cheese, dairy desserts, etc.), (2) meat and meat products (including beef, pork, poultry, among others), (3) fish and shellfish, (4) eggs, (5) cereals (i.e., rice, bread), (6) legumes (lentils, beans, etc.), (7) fruits, and (8) vegetables.

### 2.3. Sociodemographic and Anthropometric Data 

Parents or caregivers were requested to complete a survey about sociodemographic data and family backgrounds, such as parental education and occupation, family income (average monthly household income, €), employment, and lifestyle-related factors. Furthermore, parents were asked to provide general data on their children (e.g., age, sex, health status, etc.).

Information to determine if the children were eligible for inclusion in the study and for proper cohort placement was collected.

Children’s anthropometrics (height/length and weight) were given by the parents or caregivers according to the children’s health card, and then analyzed with respect to the World Health Organization sex-specific growth charts, using Anthro and Anthro Plus software (WHO Anthro for personal computers, version 3.2.2, 2011: Software for assessing growth and development of the world’s children).

### 2.4. Physical Activity and Sedentary Behavior

A detailed questionnaire modified from a previously validated questionnaire in children <10 years from Colombia was used [23]. We collected information about all of the children’s activities in 24 h for one week prior to the interview. Detailed information about the physical activity and sedentary behaviors survey is included in the article about the methodology of the EsNuPI study [18].

### 2.5. Evaluation of Plausible Reporting and Misreporting (Under- and Over-Reporting)

Subjects were identified as plausible, under-, or over-reporters of EI, considering the relationship between their basal metabolic rate and their EI using the Goldberg’s cutoffs, adapted for children. Results for the EsNuPI study have been described previously [24]. Following the EFSA recommendations [25], the non-plausible reporters were not excluded from the study sample for the present analyses because the misreporting prevalence found in the EsNuPI study was low, and the exclusion of misreporters did not result in differences in nutrient intake. However, the results of plausible reporters are shown in the Supplementary Material section.

### 2.6. Statistical Analysis

The 746 food items reported by the participants were transformed into nutrients and energy for further statistical analysis and organization of the study results.

Due to the collection of two 24-h DRs, the variance of the usual cohort intake was influenced by the individual day-to-day variation [26]. We applied the method developed by Iowa State University (ISU) in order to eradicate the intra-individual variability and to obtain an estimation of the usual intake distribution of the cohort.

The PC-SIDE software (version 1.0, Department of Statistics, Center for Agricultural and Rural Development, Ames, IA, USA) was used to implement the ISU method. This application assesses the usual nutrient intake distributions and percentiles. Whether the intake corresponds to the first 24-h DR or the telephone recall, it was considered in adjusting dietary data, stratifying by sex, age group, and cohorts (SRS or AMS).

To evaluate nutrient adequacy, the IUI was tested against the PRI for protein defined by the EFSA [4] and RDA according to IoM recommendations [2], both expressed in g protein/kg body weight/day. In order to complete the evaluation of protein adequacy, the participants were allocated by age groups established by the EFSA and IoM. We used the cutoff method based on our own reference cohort to determine the proportion of individuals who had below (<P25), between (≥P25 to ≤P75), or above (>P75) protein intake according to the distribution of our own data set by age groups.

The cutoff points were determined using data from the SRS, because this is a representative cohort (randomly selected) of the non-vegan, urban, Spanish population from one to <10 years old consuming all types of milk e.g., cow, goat, other mammal milk, and fortified and adapted milk formulas (including follow-on formula, growing up milk, toddler’s milk, and enriched and fortified milks) in the last 12 months.

To determine the normality of the sample distribution, we used the Kolmogorov–Smirnoff normality test. The Mann–Whitney U test was applied to perform comparisons by sex and age group between the cohorts (SRS and AMS).

We used the Kruskal–Wallis test to compare among age groups within cohorts. Linear correlations, collinearity tests, and logistic regressions were performed to explore any possible role of various sociodemographic, anthropometric, and physical activity variables in protein intake. The ANCOVA (analyses of covariance) was used to examine which variables could influence the intakes of total, animal, and plant protein. A probability level of 5% was accepted as the criterion for statistical significance. All calculations were made using IBM SPSS Statistics for Windows, version 20.0 (Armonk, NY, USA: IBM Corp.).

## 3. Results

### 3.1. Subjects Characteristics

The personal, anthropometric, and socioeconomic characteristics of both study cohorts by sex and age group are given in Table 1.

### 3.2. Total Protein Intake and Animal, Plant, and Mixed Protein Intakes

Table 2 shows the protein intake in grams per day (g/day), as well as animal, plant, and mixed protein intakes and protein intakes in grams per kilogram of body weight (g/kg), according to age group. Supplementary Table S1 shows the results according to age group and sex.

When comparing the AMS and SRS cohorts by age group, the children in the SRS cohort of Gp 1 had higher total protein in g/day and g/kg (47.61 vs. 43.42 g/day; 4.02 vs. 3.63 g/kg) than the same age group of the AMS cohort. The children in Gp 2 of the SRS cohort had higher total protein intake (61.67 vs. 57.35 g/day) than the peer group of the AMS cohort. Finally, the children in Gp 3 of the AMS cohort had lower animal (41.42 vs. 43.71 g/day) and plant protein (16.97 vs. 15.51 g/day) and higher intake of mixed protein (4.54 vs. 3.09 g/day) than the same age group of the SRS cohort.

Differences among age groups within the cohorts were found. In the SRS cohort, all three age groups exhibited significantly different total protein, animal, and plant protein intake (*p* < 0.001). Children of Gp 1 had significantly lower mixed protein intake than Gp 2 and Gp 3 (*p* < 0.001). For total protein in g/kg, all three age groups were statistically different (*p* < 0.001).

In the AMS cohort, all three age groups showed significant differences for total protein and mixed protein intake (*p* < 0.001), and Gp 1 showed significantly lower animal and plant protein intakes than Gp 2 and Gp 3 (*p* < 0.001). The total protein intake in g/kg from Gp 3 was significantly lower than Gp 1 and Gp 2.

Data including total, animal, plant, and mixed protein intake of the plausible reporters is included in Supplementary Table S2.

### 3.3. Contribution to Total Energy Intake from Total, Animal, Plant, and Mixed Protein

Table 3 shows the percentage of contribution of total energy from total, animal, and plant protein intakes to the total energy contribution from proteins based on two 24-h DRs of the two cohorts according to age group. Supplementary Table S3 shows the percentage of contribution to total energy from total, animal, and plant intakes from plausible reporters. The energy derived from total proteins contributed to 16.60% in the SRS and 15.49% in the AMS to the total EI. Animal protein was the main contributor (67.70% in the SRS and 68.45% in the AMS) to total protein intake, whereas the mean plant protein amounted to 25.65% and 25.20%, respectively. In addition, the mixed protein provides 6.76% in the SRS cohort and 6.36% in the AMS cohort.

Furthermore, when comparing between cohorts and age groups, the children of Gp 1 and Gp 2 of the SRS cohort had a higher percentage of contribution to the total EI from total protein (Gp 1: 15.94% vs. 14.72%; Gp 2: 16.94% vs. 15.59%, *p* < 0.005) than their peers’ groups of the AMS cohort. The children of Gp 3 in the AMS cohort had a higher contribution of mixed protein (1.08% vs. 0.76%, *p* = 0.004) and a lower contribution of total protein (16.34% vs. 16.85%, *p* = 0.022) than the same age group of the SRS cohort.

Finally, Table 4 shows the socioeconomic variables that influenced the protein intake in our study population.

### 3.4. Adequacy of Protein Intake to EFSA and IoM Recommendations

Both cohorts reported a contribution to total energy from protein (16.5% SRS and 15.6% AMS) above the 15% EI recommended by the European Society for Pediatric Gastroenterology, Hepatology, and Nutrition (ESPGHAN) Committee on Nutrition for infants and toddlers [24,29]. Moreover, 12% of the SRS and 6% of the AMS had ≥20% of energy from protein that is established as a very high protein intake by the EFSA [4] and the WHO [30].

The adequacy of the minimum value for protein intake by the EFSA and IoM expressed in g/kg body weight/day revealed that no participant showed a total protein IUI below the PRI established for each age group in both cohorts.

Table 5 shows the cutoff points established for the EsNuPI study population and based on the SRS. According to these cutoff points, 45% of the AMS cohort were between the 25th and 75th percentiles, 40% were below the P25, and 15% were above the P75. Results by the three age groups are shown in Table 6.

### 3.5. Association between Total Protein, Animal Protein, Plant Protein, and the Individual Usual Intake of Total Protein and Family- and Personal-Related Factors

Table 7 and Table 8 provide the odds ratios (ORs) and confidence intervals (CIs) analyzing total protein, animal protein, plant protein intake, and the IUI of total protein relative to family and personal factors, using age group as a control variable in both cohorts (the SRS and AMS).

In the SRS cohort, living in a municipality with more than 300,000 inhabitants was associated with a lower probability of having intakes equal to or above P75 for plant protein (OR = 0.66, 95% CI: 0.47–0.93). Meanwhile, in the AMS cohort, this same fact was associated with lower probabilities of having intakes equal to or above P75 for protein IUI (OR = 0.44, 95% CI: 0.29–0.67), total protein (OR = 0.47, 95% CI: 0.32–0.69), and animal protein (OR = 0.66, 95% CI: 0.46–0.95).

In the AMS cohort, children of families with an income between €1501–2000 showed lower probabilities of having intakes equal to or above P75 for total protein (OR = 0.51, 95% CI: 0.30–0.69), and the same applied to the fact of being in a family with an income ≥€2000 for protein IUI (OR = 0.42, 95% CI: 0.21–0.84) and plant protein (OR = 0.38, 95% CI: 0.19–0.77). Finally, in this same cohort, children who drank two or more feeding bottles of milk per day showed a 62% higher likelihood of having an intake equal to or above P75 for plant protein (*p* = 0.024).

Furthermore, as a second approach, we used anthropometric parameters as the dependent variables (Z-height for age and Z-BMI for age) and the family- and personal-related factors as independent variables; we found no significant results when performing these analyses (data not shown).

### 3.6. Food Groups Contributing to Total, Animal, and Plant Protein Intake

**Figure 1:**
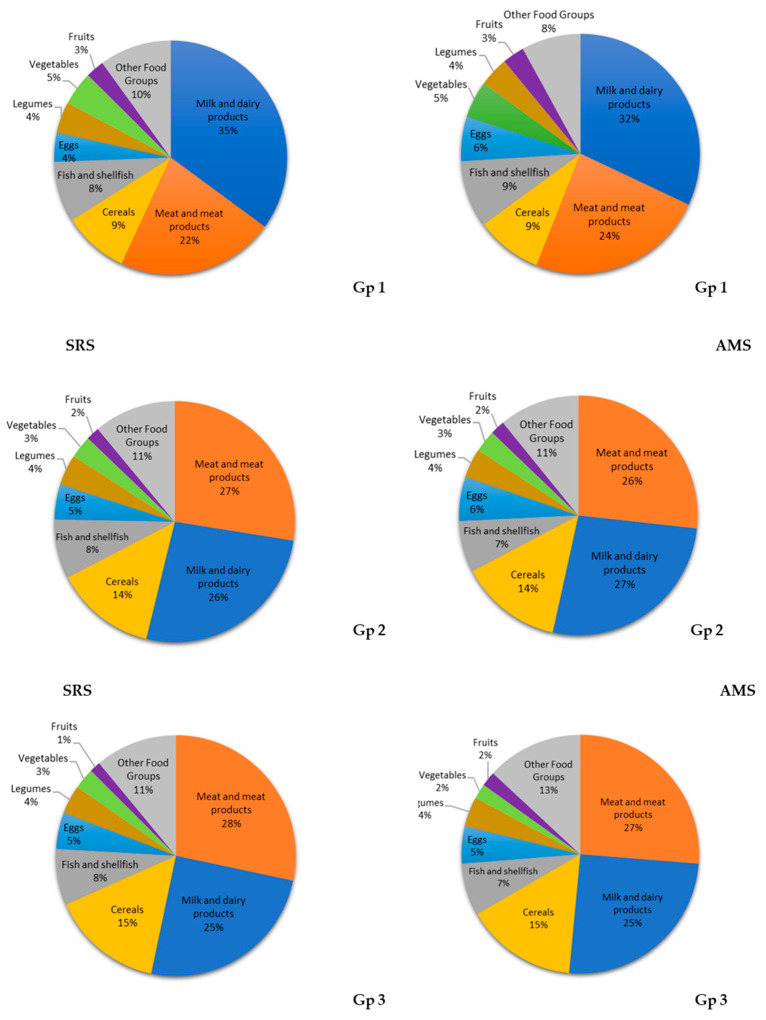
Contribution of the main eight food groups (in percentages) to total protein intake in the EsNuPI study population (Spanish reference cohort (SRS) and adapted milk consumer cohort (AMS)) according to age group (Gp 1), one to <3 years; (Gp 2), three to <6 years; and (Gp 3), six to <10 years.

The primary food sources of total protein intake in the SRS and AMS were milk and dairy products (28% and 29%), followed by meat and meat products (27% and 26%), cereals (13% and 12%, *p* = 0.012), fish and shellfish (8% and 8%), eggs (5% and 6%, *p* = 0.012), legumes (4% and 4%, *p* = 0.039), vegetables (3% and 4%), fruits (2% and 2%, *p* < 0.005), and other food groups (10% and 9%) (Figure 1).

It is important to notice that, in the SRS cohort, liquid milk (cow, goat, and other mammal milk and fortified and adapted milk formulas) provided 54.1% of the total protein intake from the food group milk and dairy products, and in the AMS cohort, the fortified and adapted milk formulas contributed 50.8% of the total protein from the same food group.

The main food sources of animal protein intake in both cohorts were the milk and dairy products (42% SRS and 43% AMS), followed by meat and meat products (39% SRS and 37% AMS, *p* = 0.043), fish and shellfish (12% SRS and 12% AMS), and eggs (7% SRS and 8% AMS, *p* = 0.026).

Regarding the plant protein intake, in both cohorts, cereals (53% SRS and 49% AMS, *p* = 0.003) were the main contributor, followed by vegetables (14% SRS and 16% AMS), legumes (13% SRS and 12% AMS, *p* = 0.045), fruits (8% SRS and 10% AMS, *p* < 0.005), and the remaining food groups (12% SRS and 13% AMS).

According to the age groups, the food groups that represented the most important sources of animal protein in the SRS and AMS cohorts of Gp 1 were milk and dairy products (49% and 46%, *p* = 0.013), meat and meat products (32% and 34%), fish and shellfish (13% and 12%), and eggs (6% and 8%, *p* = 0.044).

This order changed in groups 2 and 3 in the SRS and AMS cohorts, with meat and meat products being the first animal food sources (Gp 2: 41% and 40%; Gp 3: 42% and 40%, respectively), followed by the dairy and dairy products (Gp 2: 40% and 41%; Gp 3: 39% and 40%), fish and shellfish (Gp 2: 12% and 11%; Gp 3: 11% and 11%), and eggs (Gp 2: 7% and 8%; Gp 3: 8% and 9%).

In the three age groups from both cohorts (the SRS and AMS, respectively), the greatest contributing sources of plant protein were cereals (Gp 1: 39% and 36%; Gp 2: 54% and 55%; Gp 3: 59% and 60%), followed by the legumes (Gp 1: 13% and 11%; Gp 2: 14% and 12%; Gp 3: 12% and 12%), vegetables (Gp 1: 21% and 23%; Gp 2: 13% and 12%; Gp 3: 12% and 10%, *p* = 0.045), fruits (Gp 1: 12% and 13%, *p* = 0.036; Gp 2: 8% and 8%; Gp 3: 6% and 7%), and other food groups (Gp 1: 15% and 17%; Gp 2: 11% and 13%; Gp 3: 11% and 11%).

## 4. Discussion

This report used data from the EsNuPI study, the main objective of which is to describe food consumption, nutrient intake, and dietary patterns, as well as physical activity and sedentary behaviors in Spanish children. The present study assessed intakes of total protein and protein food sources in the two different cohorts of the EsNuPI study, both aged one to <10 years and living in urban areas, with one cohort representative of the Spanish population and one of adapted milk consumers. Furthermore, it evaluated the effect of different variables (sociodemographic, anthropometric, PAL, educational level, etc.) on protein intake.

Our results indicated differences in protein intake between the two cohorts; the SRS cohort consumed higher amounts of total protein (g/day), animal, plant, and mixed protein than the AMS cohort. On the contrary, the AMS cohort had a higher total protein (g/kg body weight/day) than the SRS.

Furthermore, both cohorts showed that no subject had an IUI of total protein intake below the PRI established by the EFSA and IoM for this range of ages.

The present study showed that the most important contributor to total protein intake among Spanish children was milk and dairy products and meat and meat products, followed by cereals, fish and shellfish, eggs, and legumes. The personal factor with more influence in the consumption of total, animal, and plant protein in both cohorts of our study was age; being between three and <10 years old seemed to be associated with the possibility of being above the median value for total, animal, and plant protein intake.

Our results showed differences between the SRS and AMS cohorts in the intakes of total, animal, plant, and mixed protein. These differences were mainly due to the diet, geographical area, level of education of the parents, and family income as the main covariates.

### 4.1. Contribution of Protein to the Total Energy and Total Protein Intake

The percentage of protein contribution to the total EI was 16.79% in the SRS cohort and 15.63% in the AMS cohort. Average total protein intakes were above the ESPGHAN (15% of the total EI) recommendations for infants and toddlers [29] and the WHO recommendations (10.0–15.0% of the total EI) [30]. It has been suggested that an excess of protein could result in an increased risk of metabolic disease later in adult life [7].

The EFSA has estimated that a very high protein intake (20% of the EI) can severely impair the water balance in children, and the extra renal water losses will be increased; therefore, high protein intakes must be avoided, especially in the first year of life [31]. The WHO concluded that, in early life, metabolic capacity to handle high protein may be less developed, and there is evidence that very high protein intakes can be harmful to health [30]. The AMDR established by the IoM for protein is 5–20% and 10–30% for children one to three years of age and four to 18 years of age, respectively [2]. In our study population, 9% in the SRS and 3% in the AMS cohorts in children one to three years exceed the 20%; moreover, no children between four and 18 years of age exceed 30%. However, the results of our study population must not be considered adequate to achieve a healthy diet. Besides, the three age groups of the SRS cohort showed a higher percentage of the contribution of protein to the total EI than the AMS cohort.

Our results are in line with a few previous studies; the ANIBES study found that EI derived from protein was 16.8%, and only 10% of the population was within the recommended range for protein intake [32]. In Flemish preschool children aged 2.5 to 6.5 years (*n* = 661) from Belgium, the contribution to the EI was 15.6% [33]. Data from NHANES 2011–2014 (*n* = 15,829) found that protein intake ranged from 13.95% to 14.25% across children aged two to eight years [12].

We evaluated the adequacy of protein intake considering children’s weight and age, and all of the EsNuPI population was above the minimum recommended intake established by the EFSA (PRI) and IoM (DRI) recommendations.

Furthermore, according to the cutoff points of our study population, the AMS cohort had less children above P75 calculated with the SRS intake data.

The contribution of protein to the EI in our study population was similar to that reported in the latest Spanish data; the ALSALMA study reported that 95.9% of children between seven and 36 months had a protein consumption more than twice the RDA [8].

Similar results were found in two groups of Irish children (*n* = 500) ages 12–24 months that consumed growing up milks (GUMs) together with cow milk (CM) or CM only. For both groups, the mean protein intake was 3.4–3.6 g/kg per day, equivalent to about three times the PRI [34].

Data from dietary surveys show that the average protein intakes in children aged one to nine years in European countries vary between 35.0 to 63 g/day. Our findings from the EsNuPI study show a mean intake of 61.4 g/day in the SRS cohort and 54.6 g/day in the AMS cohort.

Similarly, in a study of 12–24 months old French children, those drinking CM had a mean protein intake of 42 g/d, and children drinking adapted milk formula (AMF) had a protein intake of 36 g/d [35]. In the Diet and Nutrition Survey of Infants and Young Children (DNSIYC) (*n* = 2683) aged 4–18 months living in the United Kingdom, protein intakes were slightly decreased, mainly due to the decrease in CM [36]. Moreover, nutrient intake for children from the 2012 China Maternal and Infant Nutrition and Growth study (*n* = 910) aged 12 to 36 months was compared between AMF consumers and non-consumers, as well as the theoretical impact of meeting dairy intake recommendations by adding CM or AMF to children’s diets [37]. They found that, compared to the CM addition (37.4 g/day), AMF addition provided lower protein intakes (36.5 g/day).

These differences in nutrient intakes could be attributable to the differences in the composition between CM and AMF, giving rise to a lower intake of proteins in the latter. Regarding the ESPGHAN, the amount of protein in AMF available in the European market varies significantly (2.6 g/100 kcal); hence, the majority of AMF has a lower protein content than CM (4.8 g/100 kcal). In our study, children who consume AMF (the AMS cohort) have a lower intake of protein compared to the children that majorly consume CM (the SRS cohort).

The protein intake was above the RDA and PRI in both cohorts. The potential impact of high levels of protein intake on current or future health is a matter of debate and has already been discussed regarding the consequences of weight gain and renal solute load [38]. Hornell et al. published a review of children (0–18 years), and they concluded that higher protein intake during infancy is associated with increased growth, a higher BMI during childhood, and an increased risk of being overweight later in life [39]. Cerdo et al. concluded that a high intake of proteins induces a faster weight gain during infancy, which correlates with later obesity [40].

Infant protein intake, more than metabolic requirements, may stimulate the secretion of insulin-releasing amino acids (branched-chain amino acids) and insulin-like growth factor 1 (IGF-1) secretion. These factors may stimulate growth, adipogenic activity, and adipocyte differentiation. The rationale for linking increased early protein intake not only to increased weight gain, but also to the risk of obesity later in life is based on the fact that faster weight gain in infancy and childhood is associated with increased adipogenesis and later obesity risk [2,41].

Finally, in the AMS cohort, the total and plant protein intakes were associated with Z-height for age. Our results are in line with a few previous studies. In a multi-ethnic, population-based study performed in 3564 children of the Generation R Study, a higher protein intake at the age of one year was associated with a greater height, weight, and BMI up to the age of nine years [1]. In addition, the causal effect of higher protein intake in early childhood on greater weight and BMI has been confirmed in a randomized controlled trial of 1138 children comparing the impact of higher- and lower-protein formula on growth during the first year of life up to six years of age [7,42].

### 4.2. Contribution of Food Groups to Total, Animal, and Plant Protein Intake 

In the present study, the children had much higher animal protein intakes compared to other European populations [5,43,44]. However, they also had lower plant protein intake than the children of the Generation R Study at eight years old and Polish children aged six years [5,43]. Altogether, the existing dietary recommendations advise limiting animal protein intake and increasing plant-based foods to prevent chronic diseases [44].

In both cohorts, the main food sources of protein intake were similar: meat and meat products, milk and dairy products, cereals, fish and shellfish, eggs, and legumes. Milk and dairy products were the principal sources of protein across one to three years of life.

The effect of meat consumption on infant growth has been examined in various settings. As collected by the EFSA [31] in most European countries, the main contributor to dietary protein intake is meat and meat products, followed by grains and grain-based products and milk and dairy products (75% of the protein intake).

In the last few decades, in Spain, the consumption of meat and meat products has experienced great growth [45], moving away from the traditional Mediterranean diet. These changes in diet with a less varied food choice show a tendency towards an increase in the “westernized” diet [46].

Meat and meat products, milk and dairy products, fish and shellfish, and eggs were the contributors to animal protein intake in both cohorts.

Western dietary patterns high in animal sources are associated with an increased risk of metabolic syndrome [44].

Regarding the food group milk and dairy products, due to its nutritional value and macro- and micronutrient content (especially in high-quality proteins, calcium, and vitamin D), it is known that its regular consumption can be beneficial for children’s health and for proper growth and development [47,48]. According to some studies developed in Europe, children who consume more yogurt and milk also presented a healthier lifestyle; in a Spanish study, results revealed that those who consumed more milk also presented better dietary patterns [49,50].

Other important animal protein sources are fish and shellfish. Their consumption has decreased by approximately 30% in the past few years in Spain, and mainly for the youngest, which may compromise nutritional goals [51]. Moreover, the contributions from fish and shellfish to animal protein intake were higher in the EsNuPI population than in Belgium children (11% vs. 4.20%) [52].

Regarding the plant protein intake, our results show that, in both study cohorts, the main contributor was cereals and legumes. The cereals provide less protein in 100 g of the edible portion (5 to 13 g) in comparison to legumes (5 to 24 g) [20].

Similarly, in 661 Flemish preschoolers aged 2.5–6.5 years, cereals contributed most to the plant protein intake (41%). Regarding the legumes, this is one of the food groups for which intakes have negatively decreased in the last decades in Spain [52]. The high intake of legumes and cereals as major plant protein sources was associated with a lower risk of all cause and cardiovascular disease mortality [53].

Evidence has shown that plant protein not only improves body composition, but also results in lower body weight compared to animal protein [44]. Furthermore, plant protein can stimulate metabolic lipid, resulting in a better blood profile [54,55]. Günther et al. observed that a higher plant protein intake at the age of 5–6 years was associated with a lower body fat percentage [56].

More efforts are necessary to reduce the excessive amount of protein consumed by Spanish children and to redistribute the animal to plant protein ratio. The proteins from animal and plant sources have different combinations of amino acids, and the quality of the dietary protein source also necessarily influences other macro- and micronutrients in the diet [57].

### 4.3. Strength and Limitations

The present research has several strengths. The main strength is that this is the first study to analyze a representative cohort of Spanish children aged one to <10 years, an age group for which there is little information, in addition to a cohort of children of the same age consuming adapted milk. Furthermore, it is the first research evaluating total, animal, and plant protein intakes in Spanish children at this age.

The present study also has some limitations. First, bias in the reported information could have influenced the results of the study questionnaires. Hence, in line with the EFSA recommendations, under- and over-reporting were identified in this study and analyzed separately. Second, the method of two days of 24-h DR represents only the individual child’s short-term daily intake rather than usual intake. In order to decrease the influence of such a limitation, nutrient intakes were corrected for within-person variability by applying the Nusser method.

The fact that we analyzed children living in urban areas, but not in rural areas could be considered as a potential limitation. However, 52.6% of the total Spanish population from one to <10 years old live in urban areas. Moreover, the children’s weight and height were reported using their health card, and this could have generated a small gap in the calculation of the protein intake recommendations.

Future research is needed to evaluate the socioeconomic status, and more specifically, household income in the EsNuPI population, because these factors might play a role in children’s dietary preferences and choices of food quality [32].

## 5. Conclusions

In this study, we observed that protein intake in Spanish children aged one to <10 years old was above the PRI and DRI for European and international recommendations, as well as the recommended percentages for energy intakes.

Furthermore, milk and dairy products were the most important contributors to total protein intake (~50% of this contribution comes from milk), followed by meat and meat products, cereals, fish and shellfish, eggs, and legumes. Food sources were mainly of animal origin (milk and dairy products, meat and meat products, fish and shellfish, etc.) rather than plant origin (cereals, legumes, vegetables, and fruits).

Future studies should investigate the long-term effects of dietary protein in early childhood on growth and body composition, and whether high protein intake affects health later in life. Furthermore, future research should consider the quality and source of the protein in combination with the absolute amount. The findings of our study should be considered in the development and implementation of future public health policies to help establish healthy food habits and improve dietary behaviors in children.

## Figures and Tables

**Table 1:** Data on personal, anthropometric, and socioeconomic characteristics by sex and age group in the Spanish Pediatric Population (EsNuPI) study.

		Spanish Reference Cohort (SRS)			Adapted Milk Consumers Cohort (AMS)		
		Total	Boys	Girls	Total	Boys	Girls
		*n* = 707	*n* = 357	*n* = 350	*n* = 741	*n* = 371	*n* = 370
Age, mean ± SD (years)	One to <3 years	1.52 ± 0.50	1.60 ± 0.49	1.44 ± 0.50	1.46 ± 0.50	1.44 ± 0.50	1.48 ± 0.50
	Three to <6 years	3.87 ± 0.82	3.85 ± 0.82	3.89 ± 0.83	3.79 ± 0.82	3.81 ± 0.83	3.76 ± 0.82
	Six to <10 years	7.60 ± 1.12	7.55 ± 1.11	7.66 ± 1.12	7.57 ± 1.10	7.61 ± 1.11	7.53 ± 1.09
	One to <3 years	162 (22.9) *	84 (23.5) *	78 (22.3) *	294 (39.7) *	144 (38.8) *	150 (40.5) *
Age group, *n* (%)	Three to <6 years	244 (34.5) *	122 (34.2) *	122 (34.9) *	262 (35.4) *	128 (34.5) *	134 (36.2) *
	Six to <10 years	301 (42.6) *	151 (42.3) *	150 (42.9) *	185 (25) *	99 (26.7) *	86 (23.2) *
Anthropometric characteristics, median (IQR)	Z-BMI/Age	0.6 (−0.3–1.5)	0.6 (−0.3–1.5)	0.6 (−0.3–1.4)	0.5 (−0.3–(−1.4)	0.45 (−0.3–1.4)	0.5 (−0.3–1.4)
	Z-Weight/Age	0.5 (−0.3–1.2)	0.4 (−0.4–1.2)	0.6 (−0.3–1.3)	0.6 (−0.3–1.4)	0.6 (−0.1–1.4)	0.5 (−0.3–1.4)
	Z-Height/Age	−0.3 (−1.2–(−0.9))	−0.2 (−1.1–1.0)	−0.4 (−1.3–0.7)	−0.4 ** (−1.4–0.6)	−0.4 ** (−1.4–0.6)	−0.4 (−1.5–0.6)
PAL, median (IQR)	One to <3 years	1.6 (1.3–1.8)	1.6 (1.4–1.8)	1.5 (1.3–1.8)	1.5 (1.3–1.7)	1.5 (1.3–1.8)	1.5 (1.3–1.7)
	Three to <6 years	1.6 (1.4–1.7)	1.6 (1.4–1.7)	1.5 (1.4–1.7)	1.5 (1.4–1.7)	1.5 (1.4–1.7)	1.5 (1.4−1.7)
	Six to <10 years	1.6 (1.4–1.7)	1.6 (1.4–1.8)	1.6 (1.5–1.7)	1.6 (1.5–1.7)	1.6 (1.5–1.8)	1.6 (1.5−1.7)
Size of the municipality, *n* (%)	50,001 to 300,000 people	376 (53.2)	193 (54.1)	183 (52.3)	406 (54.8)	204 (55.0)	202 (54.6)
	>300,000 people	331 (46.8)	164 (45.9)	167 (47.7)	335 (45.2)	167 (45.0)	168 (45.4)
Highest level of education achieved by one of the parents, *n* (%)	≤10 years of education	23 (3.3)	10 (2.9)	13 (3.8)	14 (1.9)	7 (1.9)	7 (1.9)
	Secondary education	416 (60.5)	219 (62.9)	197 (57.9)	414 (57.0)	208 (57.5)	206 (56.6)
	University studies	249 (36.2)	119 (34.2)	130 (38.2)	298 (41.0)	147 (40.6)	151 (41.5)
Family income, *n* (%)	Low (<1500 €)	171 (24.2)	79 (22.1)	92 (26.3)	163 (22.0)	84 (22.6)	79 (21.4)
	Medium (1501 to 2000 €)	126 (17.8)	67 (18.8)	59 (16.9)	134 (18.1)	64 (17.3)	70 (18.9)
	High (>2000 €)	226 (32.0)	123 (34.5)	103 (29.4)	238 (32.1)	110 (29.6)	128 (34.6)
	No answer/doesn’t know	184 (26.0)	88 (24.6)	96 (27.4)	206 (27.8)	113 (30.5)	93 (25.1)
Number of feeding bottles or glasses of milk per day, *n* (%)	Less than two	222 (32.9)	110 (32.0)	115 (33.8)	178 (24.1)	92 (24.9)	86 (23.3)
	Two or more	459 (67.1)	234 (68.0)	225 (66.2)	561 (75.9)	278 (75.1)	283 (76.7)

Adapted from [27,28]. BMI: body mass index; IQR: interquartile range; PAL: physical activity level. The PAL was calculated according to the European Food Safety Authority (EFSA) protocol. Chi-square and Mann–Whitney tests were used to evaluate differences by total and by sex between the SRS and AMS (significant differences are marked with an asterisk (*)) and a *p*-value < 0.05 was considered statistically significant. ** *p* < 0.01 difference vs. reference cohort (Mann–Whitney’s U test).

**Table 2:** Total, animal, plant, and mixed protein intakes by age and cohort from the Nutritional Study in the Spanish Pediatric Population (EsNuPI) (*n* = 1448).

**Spanish Reference Cohort (SRS)**													
	**One to <3 years** ***n* = 162**				**Three to <6 years** ***n* = 244**				**Six to <10 years** ***n* = 301**				
**Protein (g/d)**	**Mean**	**SD**	**Median**	**IQR**	**Mean**	**SD**	**Median**	**IQR**	**Mean**	**SD**	**Median**	**IQR**	***p***
Total	48.67	16.57	47.61 ^a^	20.75	61.91	15.85	61.67 ^b^	18.66	67.85	16.69	67.04 ^c^	23.58	<0.001
Animal	33.79	12.65	33.71 ^a^	17.95	41.67	13.71	40.57 ^b^	17.29	44.79	13.42	43.71 ^c^	18.23	<0.001
Plant	11.88	6.32	10.65 ^a^	9.73	15.30	6.22	14.25 ^b^	9.30	17.64	7.07	16.97 ^c^	9.23	<0.001
Mixed ^‡^	2.63	4.38	0.87 ^a^	3.09	4.33	4.94	2.35 ^b^	6.14	5.03	5.23	3.09 ^b^	6.97	<0.001
Animal: Plant ratio	3.74	3.59	2.92	2.38	3.20	1.81	2.89	1.99	2.96	1.47	2.59	1.78	0.111
Total Protein (g/kg) ^§^	4.08	1.30	4.02 ^a^	1.66	3.62	1.16	3.49 ^b^	1.34	2.53	0.83	2.42 ^c^	1.04	<0.001
**Adapted Milk Consumer Cohort (AMS)**													
	**One to <3 years** ***n* = 294**				**Three to <6 years** ***n* = 262**				**Six to <10 years** ***n* = 185**				
**Protein (g/d)**	**Mean**	**SD**	**Median**	**IQR**	**Mean**	**SD**	**Median**	**IQR**	**Mean**	**SD**	**Median**	**IQR**	***p***
Total	44.57	15.00	43.42 *^a^	19.12	58.59	15.06	57.35 *^b^	21.64	64.81	16.21	64.44 ^c^	21.39	<0.001
Animal	31.78	12.64	30.41 ^a^	17.76	39.43	12.29	38.38 ^b^	18.95	42.32	14.14	41.42 *^b^	18.97	<0.001
Plant	10.73	4.94	9.72 ^a^	5.89	14.69	5.89	13.55 ^b^	8.36	15.96	5.80	15.51 *^b^	8.07	<0.001
Mixed ^‡^	1.65	2.77	0.70 ^a^	1.96	3.98	4.93	2.10 ^b^	4.54	6.18	6.05	4.54 *^c^	8.23	<0.001
Animal: Plant ratio	3.44	1.91	2.94	2.01	3.13	1.95	2.70	1.93	3.00	1.45	2.67	1.81	0.033
Total Protein (g/kg) ^§^	3.84	1.38	3.63 *^a^	1.70	3.60	1.08	3.52 ^a^	1.46	2.53	0.89	2.41 ^b^	1.16	<0.001

Data are presented in grams as the average intake values from two 24-h DRs, and expressed as the mean, standard deviation (SD), median, and interquartile range (IQR). ^‡^ Mixed protein from the following sources: bakery and pastry, chocolate, ready to cook/eat, appetizers, and sauces. ^§^ Mean weight of the SRS cohort was 20.8 kg, and in the AMS cohort, it was 17.4 kg. The Mann–Whitney U-test was performed to analyze differences by age group and between the SRS and AMS (significant differences are indicated using an asterisk (*) symbol following median values in the AMS cohort). Differences among age groups within the cohorts were established using the Kruskal–Wallis test (differences with statistical significance are identified using superscript letters following the median values of each age group). The *p*-values for this test are included in the last column. A *p*-value < 0.05 was considered statistically significant.

**Table 3:** Percentage of contribution of total protein and animal and plant protein intakes to the total energy intake based on two 24-h DRs of two cohorts of the Nutritional Study in the Spanish Pediatric Population (EsNuPI), according to age group (*n* = 1448).

	**Spanish Reference Cohort (SRS)**																
	**Total** ***n* = 707**				**One to <3 years** ***n* = 162**				**Three to <6 years** ***n* = 244**				**Six to <10 years** ***n* = 301**				
**Contribution (%) total protein**	**Mean**	**SD**	**Median**	**IQR**	**Mean**	**SD**	**Median**	**IQR**	**Mean**	**SD**	**Median**	**IQR**	**Mean**	**SD**	**Median**	**IQR**	***p***
Total (% EI)	16.79	2.76	16.60	3.49	15.91	3.00	15.94 ^a^	3.99	17.01	2.53	16.94 ^b^	3.37	17.09	2.70	16.85 ^b^	3.40	<0.001
Animal	67.70	11.57	68.65	15.63	70.41	12.94	72.33 ^a^	16.31	67.40	11.17	68.47 ^b^	15.13	66.24	10.86	67.65 ^b^	14.72	<0.001
Plant	25.65	9.81	24.57	12.23	24.74	11.47	23.82	13.25	25.46	9.37	23.34	12.39	26.29	9.16	25.60	11.76	0.136
Mixed ^‡^	6.76	7.80	3.65	9.47	4.85	7.45	1.64 ^a^	6.98	7.13	8.05	4.06 ^b^	9.88	7.47	9.97	4.67 ^b^	7.62	<0.001
	**Adapted Milk Consumer Cohort (AMS)**																
	**Total** ***n* = 741**				**One to <3 years** ***n* = 294**				**Three to <6 years** ***n* = 262**				**Six to <10 years** ***n* = 185**				
**Contribution (%) total protein**	**Mean**	**SD**	**Median**	**IQR**	**Mean**	**SD**	**Median**	**IQR**	**Mean**	**SD**	**Median**	**IQR**	**Mean**	**SD**	**Median**	**IQR**	***p***
Total (% EI)	15.63	2.60	15.49 *	3.19	14.92	2.46	14.72 *^a^	2.91	15.79	2.58	15.59 *^b^	3.10	16.55	2.53	16.34 *^c^	3.09	<0.001
Animal	68.45	11.31	69.76	15.14	71.29	11.13	72.56 ^a^	13.55	67.66	10.81	68.29 ^b^	15.53	65.04	11.22	66.25 ^b^	14.28	<0.001
Plant	25.20	8.81	24.42	11.89	24.93	9.44	24.30	12.22	25.64	8.74	24.84	12.66	25.01	7.83	24.48	11.12	0.504
Mixed ^‡^	6.36	7.98	3.39	8.67	3.78	6.00	1.67 ^a^	4.71	6.70	7.94	3.86 ^b^	7.65	9.95	9.27	7.07 *^c^	13.30	<0.001

DR: Dietary recall; EI: energy intake. Results are expressed as the mean, standard deviation (SD), median, interquartile range (IQR), and the percentage of contribution to the total energy intake. ^‡^ Mixed protein from the following sources: bakery and pastry, chocolate, ready to cook/eat, appetizers, and sauces. The Mann–Whitney U-test was performed to test differences by total and age group between the SRS and AMS (differences are identified with an asterisk (*) following the median values of the AMS cohort). Differences among age groups within cohorts were established using the Kruskal–Wallis test (differences with statistical significance are identified using superscript letters following the median values of each age group). The *p*-values for this test are included in the last column. A *p*-value < 0.05 was considered statistically significant.

**Table 4:** Relationship between total protein intake and socioeconomic variables in the two cohorts of the Nutritional Study in the Spanish Pediatric Population (EsNuPI) (*n* = 1448).

	Spanish Reference Cohort (SRS)				Adapted Milk Consumers Cohort (AMS)			
	Total *n* = 687				Total *n* = 726			
(g/Day)	Mean	β	CI (95%)	*p*	Mean	β	CI (95%)	*p*
**Protein**	61.45				54.62			
Geographical area (Nielsen area)		−0.486	(−1.037)–0.066	0.084		−0.111	(−0.620)–0.399	0.670
Family income		0.790	(−0.486)–2.066	0.224		1.695	0.479–2.910	0.006 *
Highest level of education achieved by one of the parents		−0.063	(−1.033)–0.907	0.898		−1.082	(−2.006)–(−0.158)	0.022 *
**Animal Protein**	41.21				37.17			
Geographical area (Nielsen area)		0.103	(−0.330)–0.535	0.641		0.028	(−0.370)–0.427	0.889
Family income		0.188	(−0.813)–1.189	0.713		1.277	0.328–2.227	0.008 *
Highest level of education achieved by one of the parents		−0.090	(−0.851)–0.672	0.817		−0.784	(−1.506)–(−0.063)	0.033 *
**Plant Protein**	15.53				13.41			
Geographical area (Nielsen area)		−0.250	(−0.459)–(−0.040)	0.019 *		−0.081	(−0.253)–0.091	0.355
Family income		0.353	(−0.132)–0.837	0.153		0.453	0.043–0.863	0.031 *
Highest level of education achieved by one of the parents		0.220	(−0.148)–0.588	0.241		−0.253	(−0.565)–0.059	0.112
**Mixed Protein ^‡^**	4.24				3.63			
Geographical area (Nielsen area)		−0.377	(−0.529)–(−0.224)	0.000 *		−0.163	(−0.306)–(−0.020)	0.026 *
Family income		0.220	(−0.133)–(0.573)	0.222		−0.019	(−0.361)–0.322	0.913
Highest level of education achieved by one of the parents		−0.204	(−0.473)–(0.064)	0.136		−0.021	(−0.281)–0.238	0.872

Nielsen area: The name given to geographical areas of Spain, with relatively homogeneous marketing characteristics, in which the commercial research company AC Nielsen divides and studies the Spanish territory. ^‡^ Mixed protein from the following sources: bakery and pastry, chocolate, ready to cook/eat, appetizers, and sauces. Data on the results are expressed as the mean, beta standardized coefficient (β), and confidence interval (CI) (95%), and *p*-values < 0.05 were considered statistically significant (significant differences are identified with an asterisk (*)). Estimation of the parameters were obtained using a covariance analysis. Protein intake (dependent variable), socioeconomic variables (covariates).

**Table 5:** Calculated protein cutoff points for the Nutritional Study in the Spanish Pediatric Population (EsNuPI) (g/d).

IUI Protein Percentiles	Total	One to <3 years	Three to <6 years	Six to <10 years
<P25	53.60	40.50	55.49	60.80
>P75	71.31	54.61	68.83	75.71

IUI: Individual usual intakes. Protein cutoff values were calculated according to the P25 and P75 of the IUI for total protein intake (expressed in g/d) in the Spanish reference cohort and by age group.

**Table 6:** Percentage of children with a below P25, between P25 and P75, and above P75 of the individual usual intake of protein in the adapted milk consumers cohort (*n* = 741).

Adapted Milk Consumer Cohort (AMS)								
	Total *n* = 741		One <3 Years *n* = 294		Three <6 Years *n* = 262		Six <10 Years *n* = 185	
IUI Protein	*n*	%	*n*	%	*n*	%	*n*	%
<25th percentile	299	40.35	117	39.80	117	44.66	65	35.14
≥25th to ≤75th percentile	335	45.21	139	47.28	110	41.98	86	45.49
>75th percentile	107	14.44	38	12.93	35	13.36	34	18.38

IUI: Individual usual intakes. Results are expressed in percentages (%). Percentiles were calculated based on the Spanish reference cohort and by age group.

**Table 7:** Odds ratios and 95% confidence intervals for intake equal to or higher than the 75th percentile (P75) for total protein IUI, total protein, and animal and plant protein relative to family and personal factors in the reference cohort of the Nutritional Study in the Spanish Pediatric Population (EsNuPI) (*n* = 707).

Spanish Reference Cohort (SRS)													
		IUI Protein (g/day) ≥P75 † (ref.: <P75)			Total Protein (g/day) ≥P75 † (ref.: <P75)			Animal Protein (g/day) ≥P75 † (ref.: <P75)			Plant Protein (g/day) ≥P75 † (ref.: <P75)		
Factor	Subcategories	OR	CI	*p*	OR	CI	*p*	OR	CI	*p*	OR	CI	*p*
Sex	Boys	1			1			1			1		
	Girls	1.20	0.86–1.69	0.287	1.32	0.94–1.86	0.113	1.28	0.91–1.80	0.158	1.32	0.94–1.86	0.113
Number of feeding bottles or glasses of milk per day	Less than two	1			1			1			1		
	Two or more	1.17	0.81–1.68	0.407	1.13	0.78–1.62	0.520	1.05	0.73–1.52	0.790	1.20	0.83–1.73	0.338
PAL	≥P50 by sex and age	1.19	0.85–1.67	0.318	1.19	0.85–1.67	0.317	1.30	0.93–1.83	0.129	0.73	0.52–1.03	0.075
Size of municipality (*n*)	50,000–300,000	1			1			1			1		
	>300,000	0.75	0.53–1.05	0.095	0.77	0.55–1.08	0.134	0.90	0.64–1.26	0.530	0.66	0.47–0.93	0.018 *
Family income (€)	≤1500	1			1			1			1		
	1501–2000	1.06	0.64–0.75	0.809	0.97	0.59–1.59	0.892	0.95	0.58–1.54	0.823	1.18	0.70–1.97	0.540
	≥2000	0.76	0.43–1.36	0.356	0.72	0.40–1.27	0.253	0.82	0.48–1.41	0.467	1.51	0.88–2.60	0.135
	Not known/no answer	1.79	1.14–2.79	0.011 *	1.65	1.06–2.56	0.027 *	1.13	0.73–1.77	0.578	1.95	1.23–3.10	0.005 *
Highest level of education achieved by one parent	≤10 years of education	1			1			1			1		
	Secondary education	0.86	0.53–1.40	0.553	0.83	0.51–1.34	0.436	0.78	0.48–1.28	0.321	0.86	0.54–1.39	0.546
	University studies	1.15	0.78–1.70	0.492	1.06	0.72–1.56	0.772	1.14	0.77–1.68	0.509	0.86	0.58–1.27	0.452

IUI, individual usual intakes; OR, odds ratio; CI, confidence intervals; PAL, physical activity level. † P75 was calculated in the reference cohort for proteins by age group and then used to categorize children according to whether their intakes were below or above this cutoff point. The age group was used as the control variable in the analyses. * A *p*-value < 0.05 was considered statistically significant.

**Table 8:** Odds ratios and 95% confidence intervals for intake equal to or higher than the 75th percentile (P75) for total protein IUI, total protein, and animal and plant protein relative to family and personal factors in the adapted milk consumer cohort of the Nutritional Study in the Spanish Pediatric Population (EsNuPI) (*n* = 741).

Adapted Milk Consumers Cohort (AMS)													
		IUI Protein (g/Day) ≥P75 † (ref.: <P75)			Total Protein (g/Day) ≥P75 † (ref.: <P75)			Animal Protein (g/day) ≥P75 † (ref.: <P75)			Plant Protein (g/day) ≥P75 † (ref.: <P75)		
Factor	Subcategories	OR	CI	*p*	OR	CI	*p*	OR	CI	*p*	OR	CI	*p*
Sex	Boys	1			1			1			1		
	Girls	0.81	0.53–1.22	0.304	0.92	0.63–1.35	0.677	0.70	0.49–1.02	0.062	1.19	0.81–1.75	0.387
Number of feeding bottles or glasses of milk per day	Less than two	1			1			1			1		
	Two or more	1.35	0.85–2.12	0.202	1.45	0.95–2.21	0.084	0.98	0.63–1.50	0.910	1.62	1.07–2.47	0.024 *
PAL	≥P50 by sex and age	1.29	0.85–1.94	0.226	1.38	0.94–2.02	0.098	1.22	0.85–1.76	0.286	1.09	0.74–1.60	0.675
Size of municipality (*n*)	50,000–300,000	1			1			1			1		
	>300,000	0.44	0.29–0.67	<0.001 *	0.47	0.32–0.69	<0.001 *	0.66	0.46–0.95	0.027 *	0.73	0.49–1.07	0.109
Family income (€)	≤1500	1			1			1			1		
	1501–2000	0.60	0.33–1.07	0.085	0.51	0.30–0.88	0.016 *	0.75	0.45–1.25	0.271	0.56	0.31–1.01	0.055
	≥2000	0.42	0.21–0.84	0.014 *	0.42	0.23–0.78	0.006 *	0.66	0.38–1.15	0.146	0.38	0.19–0.77	0.007 *
	Not known/no answer	0.76	0.46–1.25	0.287	0.65	0.41–1.03	0.068	0.67	0.42–1.06	0.088	1.21	0.76–1.92	0.417
Highest level of education achieved by one parent	≤10 years of education	1			1			1			1		
	Secondary education	1.21	0.67–2.21	0.526	1.14	0.65–1.99	0.650	1.37	0.82–2.29	0.228	0.75	0.40–1.41	0.374
	University studies	1.54	0.97–2.45	0.065	1.57	1.03–2.40	0.038 *	1.34	0.89–2.03	0.160	1.53	0.99–2.35	0.055

IUI, individual usual intakes; OR, odds ratio; CI, confidence intervals; PAL, physical activity level. † P75 was calculated in the reference cohort for proteins by age group and then used to categorize children in the AMS according to whether their intakes were below or above this cutoff point. The age group was used as the control variable in the analyses. * A *p*-value < 0.05 was considered statistically significant.

## Supplementary Materials

The following are available online at https://www.mdpi.com/2072-6643/13/4/1062/s1, Table S1: Total, animal, plant, and mixed protein intakes by age and cohort from the Nutritional Study in Spanish Pediatric Population (EsNuPI), according to age group and sex (*n* = 1448), Table S2: Total protein intake based on two 24 h dietary recalls from plausible reporters of the Nutritional Study in Spanish Pediatric Population (EsNuPI), according to age group and cohort (*n* = 1216), Table S3: Percentage of contribution of total protein, animal and plant protein intakes to the total energy intake based on two 24 h DR from plausible reporters of two cohorts of Nutritional Study in Spanish Pediatric Population (EsNuPI), according to age group (*n* = 1216).
