# Supplementary material for: Dietary Intake, Nutritional Adequacy, and Food Sources of Protein and Relationships with Personal and Family Factors in Spanish Children Aged One to <10 Years: Findings of the EsNuPI Study
Source: Nutrients. 2021 Mar 24;13(4):1062. doi: 10.3390/nu13041062 (PMC8064310; doi:10.3390/nu13041062)
Supplement: Supplementary file 1 [file nutrients-13-01062-s001.pdf]

Supplementary Table S1. Total, animal, plant, and mixed protein intakes by age and cohort from the Nutritional Study in Spanish Pediatric Population (EsNuPI), according to age group and sex (n = 1448).

	**Spanish Reference Cohort (SRS)**											
	**Total**			**1 to <3 years**			**3 to <6 years**			**6 to <10 years**		
	**Total**	**Boys**	**Girls**	**Total**	**Boys**	**Girls**	**Total**	**Boys**	**Girls**	**Total**	**Boys**	**Girls**
**Protein (g/d)**	707	357	350	162	84	78	244	122	122	301	151	150
**Total**	60.89	64.42	60.35	47.61	48.24	47.03	61.67	64.73	58.88	67.04	69.72	65.22
**Animal**	40.75	42.42	39.34	33.71	33.79	33.52	40.57	42.97	39.54	43.71	44.68	42.00
**Plant**	14.77	14.85	14.71	10.65	11.81	10.96	14.25	14.40	14.14	16.97	18.07	16.36
**Mixed** ‡	2.10	2.10	2.24	0.87	0.58	0.98	2.35	2.12	2.38	3.09	3.10	3.08
**Animal: Plant ratio**	2.78	2.80	2.74	2.92	2.90	2.94	2.89	2.87	2.89	2.59	2.61	2.55
**Total Protein (g/kg)** §	3.07	3.12	3.04	4.02	3.92	4.23	3.49	3.45	3.52	2.42	2.53	2.35
	**Adapted Milk Consumers Cohort (AMS)**											
	**Total**			**1 to <3 years**			**3 to <6 years**			**6 to <10 years**		
	**Total**	**Boys**	**Girls**	**Total**	**Boys**	**Girls**	**Total**	**Boys**	**Girls**	**Total**	**Boys**	**Girls**
**Protein (g/d)**	741	371	370	294	144	150	262	128	134	185	99	86
**Total**	53.43	54.40*	54.75*	43.42	42.31*	44.28	57.35	55.65*	58.63	64.44	65.95	62.81
**Animal**	36.05	35.76*	36.24*	30.41	29.40*	31.53	38.38	37.15*	40.24	41.42	41.53	40.96
**Plant**	12.46	12.11*	12.86*	9.72	9.16	10.22	13.55	13.88	13.10	15.51	15.32	15.52
**Mixed** ‡	1.83	1.95	1.75*	0.70	0.67	0.92	2.10	2.39	1.95	4.54	4.31	4.71
**Animal: Plant ratio**	2.79	2.80	2.75*	2.94	3.12	2.83	2.70	2.67	2.72	2.67	2.67	2.65
**Total Protein (g/kg)** §	3.26	3.18	3.43*	3.63	3.53	3.75	3.52	3.40	3.62	2.41	2.43	2.35

Data presented in grams as the average intake values from two 24-h DR. ‡ Mixed protein from the following sources: bakery and pastry, chocolate, ready to cook/eat, appetizers and sauces. § Mean weight of SRS cohort was 20.8 kg and in the AMS cohort was 17.4 kg. Results are expressed as median. Mann–Whitney U-test was performed to analyze differences by total and sex between SRS and AMS (significant differences are indicated using an asterisk (*) symbol following median values in the AMS cohort). p-value <0.05 was considered statistically significant.

Supplementary Table S2. Total protein intake based on two 24-h dietary recalls from plausible reporters of the Nutritional Study in Spanish Pediatric Population (EsNuPI), according to age group and cohort (n = 1216).

**Spanish Reference Cohort (SRS)**																	
	**Total** *n* = 598				**1 to <3 years** *n* = 120				**3 to <6 years** *n* = 211				**6 to <10 years** *n* = 267				
**Protein (g/d)**	**Mean**	**SD**	**Median**	**IQR**	**Mean**	**SD**	**Median**	**IQR**	**Mean**	**SD**	**Median**	**IQR**	**Mean**	**SD**	**Median**	**IQR**	***p***
**Total**	61.72	17.06	61.32	22.48	46.20	14.43	45.37[a]	16.88	62.01	14.16	60.81[b]	17.84	68.53	15.69	67.87[c]	21.55	<0.001
**Animal**	41.39	13.67	40.77	17.49	32.21	11.78	32.60[a]	17.91	41.91	12.68	41.03[b]	16.48	45.10	13.35	44.12[c]	18.32	<0.001
**Plant**	15.71	6.85	15.01	9.69	11.36	6.23	10.09[a]	9.05	15.32	6.14	14.29[b]	9.14	17.98	6.66	17.56[c]	9.03	<0.001
**Mixed** ‡	4.18	4.83	2.12	5.97	2.13	3.56	0.87[a]	2.36	4.27	4.73	2.35[b]	5.71	5.03	5.14	3.15[b]	7.05	<0.001
**Animal: Plant ratio**	3.18	2.15	2.71	1.91	3.76	3.51	2.90	2.49	3.23	1.83	2.90	1.91	2.88	1.40	2.56	1.78	0.048
**Total Protein (g/kg)** §	3.16	1.04	3.05	1.41	3.83	1.04	3.90[a]	1.28	3.58	0.95	3.48[b]	1.20	2.53	0.69	2.48[b]	0.94	<0.001
**Adapted Milk Consumers Cohort (AMS)**																	
	**Total** *n* = 618				**1 to <3 years** *n* = 236				**3 to <6 years** *n* = 224				**6 to <10 years** *n* = 158				
**Protein (g/d)**	**Mean**	**SD**	**Median**	**IQR**	**Mean**	**SD**	**Median**	**IQR**	**Mean**	**SD**	**Median**	**IQR**	**Mean**	**SD**	**Median**	**IQR**	***p***
**Total**	54.10	16.52	52.81*	23.07	42.82	12.29	4258*[a]	16.80	57.47	13.32	57.01*[b]	19.99	66.18	15.50	66.22[c]	21.01	<0.001
**Animal**	3.30	1.02	3.22*	1.36	3.62	1.04	3.54*[a]	1.42	3.48	0.89	3.44[a]	1.29	2.58	0.76	2.49[b]	1.05	<0.001
**Plant**	36.82	13.19	35.82*	17.63	30.54	10.97	29.31[a]	15.79	39.00	11.68	37.82*[b]	18.01	43.12	14.29	42.41[c]	18.77	<0.001
**Mixed** ‡	13.19	5.67	12.30*	4.76	10.23	4.39	9.20[a]	5.42	14.19	5.53	12.93[b]	7.12	16.19	5.49	15.54*[c]	7.76	<0.001
**Animal: Plant ratio**	3.68	4.97	1.87*	4.76	1.67	2.87	0.70[a]	1.97	3.83	4.83	2.10[b]	4.24	6.46	6.14	4.71*[c]	8.26	<0.001
**Total Protein (g/kg)** §	3.25	1.87	2.79	1.91	3.47	1.92	2.94	2.32	3.22	2.05	2.72	1.89	2.98	1.47	2.65	1.81	0.043

Data presented in grams as the average intake values from two 24-h DR, and expressed as mean, standard deviation (SD), median, and interquartile range (IQR). ‡ Mixed protein from the following sources: bakery and pastry, chocolate, ready to cook/eat, appetizers and sauces. § Mean weight of SRS cohort was 20.8 kg and in the AMS cohort was 17.4 kg. Mann–Whitney U-test was performed to analyze differences by age group and between SRS and AMS (significant differences are indicated using an asterisk (*) symbol following median values in the AMS cohort). Differences among age groups within cohorts were established using the Kruskal–Wallis test (differences with statistical significance are identified using superscript letters following the median values of each age group). *p*-values for this test are included in the last column. *p*-value <0.05 was considered statistically significant.

Supplementary Table S3. Percentage of contribution of total protein, animal and plant protein intakes to the total energy intake based on two 24-h DR from plausible reporters of two cohorts of Nutritional Study in Spanish Pediatric Population (EsNuPI), according to age group (*n* = 1216).

	Total *n* = 598				1 to <3 years *n* = 120				3 to <6 years *n* = 211				6 to <10 years *n* = 267				
**Spanish Reference Cohort (SRS)**																	
**Contribution (%) total protein**	**Mean**	**SD**	**Median**	**IQR**	**Mean**	**SD**	**Median**	**IQR**	**Mean**	**SD**	**Median**	**IQR**	**Mean**	**SD**	**Median**	**IQR**	***p***
**Total (% EI)**	16.78	2.62	16.62	3.27	16.00	3.04	16.09[a]	4.13	16.97	2.43	16.90[b]	3.23	16.98	2.51	16.79[b]	3.05	0.013
**Animal**	67.43	11.41	68.59	15.20	70.47	13.16	72.53[a]	14.47	67.69	10.84	68.45[b]	14.66	65.85	10.73	67.38[b]	15.09	<0.001
**Plant**	25.83	9.81	24.92	12.06	25.13	11.97	24.21	14.43	25.23	9.24	22.98	12.06	26.63	9.13	25.97	11.18	0.089
**Mixed ‡**	6.74	7.69	3.66	9.03	4.39	7.02	1.64[a]	6.01	7.08	7.82	4.09[b]	9.23	7.52	7.69	4.72[b]	10.03	<0.001
**Adapted Milk Consumer Cohort (AMS)**																	
	**Total *n* = 618**				**1 to <3 years *n* = 236**				**3 to <6 years *n* = 224**				**6 to <10 years *n* = 158**				
**Contribution (%) total protein**	**Mean**	**SD**	**Median**	**IQR**	**Mean**	**SD**	**Median**	**IQR**	**Mean**	**SD**	**Median**	**IQR**	**Mean**	**SD**	**Median**	**IQR**	***p***
**Total (% EI)**	15.62	2.48	15.55*	3.10	14.89	2.50	14.78*[a]	2.99	15.90	2.38	15.77*[b]	2.89	16.33	2.31	16.10*[b]	3.02	<0.001
**Animal**	68.43	11.46	69.66	15.82	71.28	11.41	72.55[a]	14.71	68.02	10.81	68.43[b]	14.87	64.74	11.38	65.75[c]	14.57	<0.001
**Plant**	25.02	8.93	24.39	11.76	24.77	9.68	24.17	12.07	25.31	8.98	24.39	12.56	24.99	7.67	24.69	11.06	0.554
**Mixed ‡**	6.55	8.22	3.59	8.64	3.94	6.28	1.81[a]	4.81	6.66	8.05	3.86[b]	7.45	10.27	9.49	7.41*[c]	13.33	<0.001

DR: Dietary Recall; EI: energy intake. Results are expressed as mean, standard deviation (SD), median, and interquartile range (IQR), percentage of contribution to the total energy intake. ‡ Mixed protein from the following sources: bakery and pastry, chocolate, ready to cook/eat, appetizers and sauces. Mann–Whitney U-test was performed to test differences by total and age group between SRS and AMS (differences are identified with an asterisk (*) following the median values of the AMS cohort). Differences among age groups within cohorts were established using the Kruskal–Wallis test (differences with statistical significance are identified using superscript letters following the median values of each age group). *p*-values for this test are included in the last column. *p*-value < 0.05 was considered statistically significant.
